# Mechanistic Studies of the Solvolyses of Carbamoyl Chlorides and Related Reactions

**DOI:** 10.3390/ijms17010111

**Published:** 2016-01-15

**Authors:** Malcolm J. D’Souza, Dennis N. Kevill

**Affiliations:** 1 Department of Chemistry, Wesley College, Dover, DE 19901-3875, USA; 2 Department of Chemistry and Biochemistry, Northern Illinois University, DeKalb, IL 60115-2862, USA

**Keywords:** carbamoyl chloride, disubstituted and monosubstituted, Grunwald–Winstein equation, ionization reaction, isocyanate

## Abstract

Carbamoyl chlorides are important intermediates, both in the research laboratory and in industrial scale syntheses. The most studied and used are the disubstituted derivatives, incorporating either aryl or alkyl groups (Ar_2_NCOCl or R_2_NCOCl). Sometimes, the groups are tied back to give a ring and piperidino- and morpholino-derivatives are commonly encountered. Some studies have been made with two different groups attached. Solvolyses tend to occur at the carbonyl carbon, with replacement of the chloride ion. Studies of both rate and products are reviewed and the solvolysis reactions are usually S_N_1, although addition of an amine leads to a superimposable bimolecular component. Many of the studies under solvolytic conditions include the application of the extended Grunwald–Winstein equation. The monosubstituted derivatives (ArNHCOCl or RNHCOCl) are less studied. They are readily prepared by the addition of HCl to an isocyanate. In acetonitrile, they decompose to set up and reach equilibrium with the isocyanate (ArNCO or RNCO) and HCl. Considering that the structurally related formyl chloride (HOCOCl) is highly unstable (with formation of HCl + CO_2_), the unsubstituted carbamoyl chloride (H_2_NCOCl) is remarkably stable. Recommended synthetic procedures require it to survive reaction temperatures in the 300–400 °C range. There has been very little study of its reactions.

## 1. Introduction

In contrast to the parent acid of chloroformate esters (HOCOCl), where attempts to prepare it lead to carbon dioxide and hydrogen chloride [1], the corresponding parent structure for carbamoyl chlorides (H_2_NCOCl) is a stable molecule, even at quite high temperatures [2,3,4]. It can be stabilized further by complexation with aluminum chloride or iron (III) chloride [2].

**Scheme 1:**
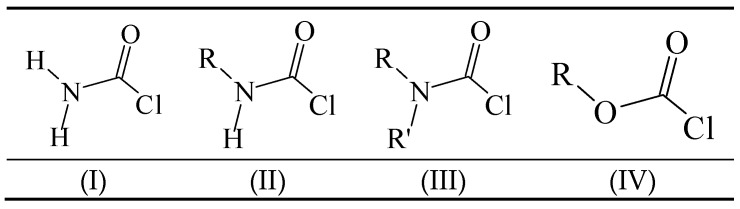
Standard structural representations for substituted carbamoyl chlorides and chloroformate esters.

The carbamoyl chlorides of types I, II, and III resemble the chloroformate (IV), with the alkoxy or aryloxy group replaced by an amino (as in I), an *N*-substituted amine (as in II), or an *N*,*N*-disubstituted amino group (as in III). In the type III carbamoyl chlorides, there is the possibility of the R and R′ being tied back to give a ring, as in piperidino- or morpholino-carbamoyl chlorides (Scheme 1).

Reactions of I, II, and III type compounds with ammonia or with primary and secondary amines leads to urea (H_2_NCONH_2_) or to derivatives of urea, with from zero to four alkyl or aryl groups replacing the four hydrogens of the urea structure. The reactions with an alcohol or phenol lead to stable carbamate esters (urethanes) and reaction with water leads initially to RR′NCOOH (R and R′ can be H, alkyl, or aryl) as a short-lived intermediate which rapidly decomposes to RR′NH, with loss of CO_2_.

An interesting historical aspect of the naming is that, within the parent carbamoyl chloride and its derivatives, it is not consistent and, especially in the older literature, “carbamyl” is often found. Indeed, within recent catalogs of fine chemicals some entries use one form and others the alternative. One recent “Handbook of Fine Chemicals” has the listing as Diphenylcarbamoyl chloride but, nearby, we find Dimethylcarbamyl chloride. In doing a computer-assisted literature search, this dichotomy makes a search by topic more difficult than necessary. This anomaly is clarified somewhat by viewing the IUPAC Rules for Organic Nomenclature as presented in the “Handbook for Chemical Society Authors,” published by the Chemical Society (London) in 1961 [5]. The naming of radicals from “amic acids” (half amides of dibasic acids) is, according to Rule 58.4 (put forward in 1951), with the ending “amoyl.” In a footnote, it is further stated that this required a change in British practice from carbamyl to carbamoyl. This explains the prevalence of the carbamyl form in the early literature. The continued use of this form must be ascribed to inertia and, possibly, to researchers following the naming used by the suppliers.

In view of the connection of carbamoyl-containing compounds to the simple natural product urea, it is not surprising that carbamoyl chlorides, or isocyanate esters formed by elimination of HCl from an *N*-substituted carbamoyl chloride, find use in the development of materials for use as herbicides [6,7], fungicides [8,9], and pesticides [10,11]. Other applications include the reaction with eseridine to give derivatives with antichlolinesterase activity [12], the preparation of phenylureas used in the development of compounds with antimycobacterial activity [13], the syntheses of dicycloalkylcarbamoyl ureas used for the enhancement of glucokinase activity [14], and the syntheses of several *N*-carboxylic acid heterocyclic derivatives with muscle relaxant and anticonvulsant properties [15].

In this review, emphasis will be on the detailed reaction pathways followed when substitution and elimination reactions of carbamoyl chlorides are operative. Such reactions are crucial steps within the synthetic applications outlined above.

The investigation of these types of reaction pathways are heavily dependent on various types of kinetic studies, frequently of solvolysis reactions. Studies of the solvolysis reactions of the closely related haloformate esters [16] and of the effects of sulfur for oxygen substitution upon those solvolyses [17] have recently been reviewed. The influence of temperature can lead to energies and entropies of activation, with entropies of activation being useful in distinguishing between unimolecular and bimolecular pathways, with the latter tending to have appreciably negative values due to two species being associated, with a restricted orientation, at the transition state [18].

Isotope effects can also be useful, with isotopic substitution within the reactants. In the case of a solvolysis reaction, substitution within the solvent is also substitution within a reactant. The kinetic solvent isotope effect (KSIE) observed on substitution of deuterium for hydrogen (*k*_H_/*k*_D_) was helpful in the studies of chloroformate esters [16], and one would expect it to be similarly helpful in the solvolyses of carbamoyl chlorides in water itself and in mixtures of water with a relatively inert organic solvent, such as acetone or dioxane, or in methanol (allowing for enhanced solubility of the substrate relative to water).

Linear free energy relationships (LFERs) can be especially useful [19,20,21]. A classical example of an LFER is the Hammett Equation [22], which can be applied to reactions in which an aromatic group is present in a reactant. Varying substituents are introduced into *meta*- and *para*- positions and their influence is correlated by the Hammett Equation (Equation (1)).

log (*k/k_o_*) = ρσ + *c* (1)

In Equation (1), *k* and *k*_o_ are the rates of reaction with and without a substituent, σ is a scale of substituent constants and ρ is the reaction constant. The sign and magnitude of ρ can be related to the reaction mechanism. The constant *c*, a residual constant, is most frequently omitted in applications of this equation.

A second LFER, applicable only to solvolysis reactions, was initially developed for application to ionization reactions within a series of solvents. A scale of solvent ionizing power *Y*, was established based on the specific rates of solvolysis of *tert*-butyl chloride [23]. It was subsequently suggested that, in principle at least, bimolecular solvolyses could be accommodated by adding a second term to the LFER, governed by the sensitivity to changes in solvent nucleophilicity [24]. It was some 25 years before such a scale was developed [25]. The same research group demonstrated that the solvolyses of *tert*-butyl chloride includes in the LFER a nucleophilic component and *Y* scales (*Y*_X_ for leaving group X) have been developed using derivatives of the adamantane cage structure [26,27,28]. The initial solvent nucleophilicity scale was based on the specific rates of solvolysis of methyl *p*-toluenesulfonate (tosylate) [25]. A subsequent scale (*N*_T_) based on the solvolyses of *S*-methyldibenzothiophenium ion is now usually employed [29,30]. A brief review [31], published in recognition of the sixty-year anniversary of the initial publication, outlines the history of the development and application of the extended (two-term) Grunwald-Winstein equation (Equation (2)).

log (*k/k_o_*)_RX_ = *lN*_T_ + *mY*_X_ + *c* (2)

In Equation (2), *k* and *k*_o_ are the specific rates of solvolysis in a given solvent and in the standard solvent (80% ethanol), *l* is the sensitivity towards changes in solvent nucleophilicity (*N*_T_), *m* is the sensitivity towards changes in solvent ionizing power *Y*_X_, and *c* is a constant (residual term). Opinions vary as to whether a *c* term should be included in correlations of this type [21,32].

Also useful can be leaving-group effects in substitution and/or elimination reactions. Application of this technique to bromide *versus* tosylate ratios (*k*_OTs_/*k*_Br_), introduced by Hoffmann [33], has been shown to be a good and widely used technique for distinguishing between unimolecular (S_N_1 + E1) and bimolecular (S_N_2 + E2) pathways, with appreciably larger ratios for unimolecular reactions [34]. Other leaving-group ratios have also been considered, such as the *k*_OTs_/*k*_Cl_ and, especially, *k*_X_/*k*_Br_. This allows the influence of a variety of leaving groups X relative to that of bromide to be tabulated for a variety of reactions [35]. For haloformate ester solvolyses, the *k*_F_/*k*_Cl_ ratio was found to be useful, with the bimolecular reaction now referring to the addition step of an addition-elimination (association-dissociation) pathway [16]. For this pathway, the values were close to unity, since the carbon-halogen bond is not broken in the rate-determining step (RDS). For the unimolecular pathway (S_N_1 + E1), the value for the ratio is very small because of the much stronger C–F bond present in the fluoroformate.

Another tool that can be useful is to determine selectivity ratios for the product portioning as a binary solvent composition is varied, usually presented as a plot against the solvent composition. If a discontinuity is seen in a region of solvent composition where other evidence also suggests a changeover in the dominant pathway, then this can be considered to give support for the proposed changeover. The product selectivities are obtained by combining measured product ratios with the concentration ratios of a binary solvent from which the product can be formed by attack of either component. For example, Bentley has made extensive use of this technique in establishing (or excluding) dual reaction channels in the solvolyses of acyl chlorides (RCOCl), and also sulfonyl chlorides (RSO_2_Cl), in alcohol-water mixtures. These solvolyses can lead to either an ester (attack by alcohol) or an acid (attack by water) [36,37,38,39].

## 2. Solvolyses of *N*,*N*-Dialkylcarbamoyl Chlorides

The first mechanistic study involving a carbamoyl chloride appears to be the study by Hall of the hydrolysis of *N*,*N*-dimethylcarbamoyl chloride [40]. He found the specific rates of hydrolysis to be considerably higher than those for ethyl chloroformate. The specific rate for hydrolysis of *N*,*N*-diethylcarbamoyl chloride was too high to measure at the 8.5 °C convenient for *N*,*N*-dimethylcarbamoyl chloride. It was found that the specific rates fell in value as the solvolysis progressed, reasonably attributed to the operation of a “mass law” effect of return of the chloride to the carbamoyl cation within the initial products of an S_N_1 process. Similarly, addition of 0.05 M pyrollidine leads to 52% of the product involving capture by pyrollidine with only a minor increase in the rate of reaction. These factors plus the observation of a positive entropy of activation of +5.6 cal·mol^−1^·K^−1^, as opposed to a value of −12.4 cal·mol^−1^·K^−1^ for ethyl chloroformate, strongly indicated a unimolecular ionization pathway for the dimethylcarbamoyl chloride hydrolysis and probably a bimolecular pathway for the ethyl chloroformate hydrolysis.

Hall and Morgan [41] succeeded in observing a bimolecular pathway for dimethylcarbamoyl chloride by studying the reactions with a modest excess of a cyclic secondary amine, 2-methylpiperidine, in the low polarity solvent benzene. They reported the progress of this reaction, and also of a parallel reaction with ethyl chloroformate, at 30.0 °C only in terms of the observed percentage completion of the reactions at a given time. This data can be converted to approximate second-order rate coefficients, first-order in each reactant, of 1.4 × 10^−3^ L·mol^−1^·s^−1^ for dimethylcarbamoyl chloride and 1.2 L·mol^−1^·s^−1^ for ethyl chloroformate. The approximately three orders of magnitude faster reaction of the chloroformate strongly suggested, as opposed to a faster S_N_1 reaction of the carbamoyl chloride in hydrolysis, that the reactions with a fairly powerful nucleophile in the low ionizing power solvent benzene are now both proceeding by a bimolecular reaction.

Several years later, Hall and Lueck [42] investigated the influence of the mercury (II) ion on the hydrolyses of several acid chlorides. They found a marked catalysis to the hydrolyses of dimethylcarbamoyl chloride, attributed to electrophilic assistance at the chloride to a reaction already favoring ionization. Essentially, no assistance was given to the *n*-butyl chloroformate hydrolysis in 50% dioxane–50% water. This parallels the behavior towards the silver (I) ion, also a powerful reagent for electrophilic assistance to removal of chloride ion from carbon [43]. In 50% acetonitrile–50% water, the rates of solvolysis of ethyl chloroformate were unchanged on adding silver perchlorate [44]. Indeed, in contrast to the reactions of alkyl halides [43], the reactions of alkyl chloroformates with silver nitrate in acetonitrile involve either attack by silver-ion (2° alkyl) [45] or by nitrate ion (methyl or 1° alkyl) [46], but not by both simultaneously.

When Hall wished to develop a scale of nucleophilic reactivity for a series of amines in ethanol as solvent, he returned to a consideration of their second-order rate coefficients for reactions with *N*,*N*-dimethylcarbamoyl chloride [47]. He found the Swain–Scott equation [20,48,49] to be more useful in this regard than the Taft equation [49], a variant of the Hammett equation [22] for use with aliphatic substrates. The equation used is shown in Equation (3) for hydrolysis, but data in a wide variety of solvents can be correlated.

log *k*_2_ − log *k*_1_/[H_2_O] = *sn* (3)

In Equation (3), *k*_2_ is the second-order rate coefficient for the substrate-amine reaction, *k*_1_ is the first-order rate coefficient for the hydrolysis of the substrate, and *s* is the sensitivity to changes in nucleophilicity (*n*).

In pioneering work on the development of an apparatus for the measurement of the specific rates of fast solvolysis by conductivity, Ugi and Beck [50] measured the rate of increase in acid concentration for solvolyses of several substrates in water-acetone mixtures at temperatures in the range of −50 to +40 °C. At −20 °C, in 89.1% acetone–10.9% water (*v*/*v*), the specific rates of solvolysis of 1-piperidineocarbonyl chloride and diisopropylcarbamoyl chloride were 2.1 × 10^−3^·s^−1^ and 8.4 × 10^−2^·s^−1^, respectively. The faster reaction with two 2° alkyl groups on the nitrogen, as opposed to the primary situation of the adjacent carbons in the piperidine ring, was considered to be supporting evidence for an S_N_1 pathway.

Consistent with the proposed unimolecular pathway, the solvolysis of *N*,*N*-diethylcarbamoyl chloride was slightly faster than that of the *N*,*N*-dimethylcarbamoyl chloride, by a factor of 4.2 in 80% ethanol at 25.0 °C and of 6.6 in 100% methanol at 25.0 °C [51].

Queen [52] made very accurate conductivity determinations at precisely controlled temperatures, in the range of 0.3–14 °C, to obtain specific rates of hydrolysis for *N*,*N*-dimethylcarbamoyl chloride, together with determinations for several chloroformate esters. The solvent used was 100% H_2_O. The fall-off in specific rate value for the hydrolysis of the carbamoyl chloride as reaction progressed [40] was confirmed, and the mass law effect was minimized by working at a very low (about 4 × 10^−4^ M) substrate concentration. Values for the activation energy of 20.63 kcal/mol and for the activation entropy of +3.50 cal·mol^−1^·K^−1^ are consistent with the less precise values of 21.0 kcal/mol, and +5.6 cal·mol^−1^·K^−1^ obtained earlier [40]. Values for the entropy of activation in the range of −19.8 to −16.6 cal·mol^−1^·K^−1^ obtained for phenyl, methyl and *n*-propyl chloroformates as opposed to the value of +10.1 cal·mol^−1^·K^−1^ for the isopropyl chloroformate, and the positive value for the *N*,*N*-dimethylcarbamoyl chloride supported the previous proposals of bimolecular and unimolecular processes [40,53].

Bacaloglu, Dăescu, and Ostrogovich [54] studied the solvolyses of *N*,*N*-dimethylcarbamoyl chloride in nine alcohols and in the highly ionizing 99% formic acid. In the same publication, they also present a study of the ethanolysis of a series of *N*,*N*-dialkylcarbamoyl chlorides. Both types of study were carried out at several temperatures and the ΔH^≠^ and ΔS^≠^ activation parameters were determined for all of the studied systems. Further, the piperidino and morpholino compounds were solvolyzed in 50% acetone, ethanol, and cyclohexanol, with activation parameters determined for the first two solvents.

While reactions of the *N*,*N*-dialkylcarbamoyl chlorides with amines in benzene or ethanol exhibit second-order kinetics and are believed to be bimolecular [41,47], solvolyses in water and aqueous acetone had been considered to be S_N_1 in nature, with one indication being a positive entropy of activation [40,52]. It is a little surprising that, in this study, of 24 entropies of activation reported, only three are positive, all for solvolyses in 50% acetone, and some are extremely negative, such as the values of −38.4 cal·mol^−1^·K^−1^ for *N*,*N*-dimethylcarbamoyl chloride solvolyzing in 2-propanol or, more surprising, the −37.8 cal·mol^−1^·K^−1^ for a solvolysis in 50% ethanol of the *N*,*N*-diisopropylcarbamoyl chloride as opposed to + 2.6 cal·mol^−1^·K^−1^ for the corresponding solvolysis of the *N*,*N*-dipropylcarbamoyl chloride. To quote the authors, “It is remarkable that some of the reactions studied are characterized by a very low activation entropy although the mechanism is unimolceular. This demonstrates that the entropy criterion of mechanism must be used carefully and in connection with other arguments.”

In the analysis of the effect of varying the alcohol in the solvolyses at 25.0 °C of the *N*,*N*-dimethylcarbamoyl chloride, they applied the original form (Equation (4)) of the Grunwald–Winstein equation [23].

log (*k/k_o_*) = *mY* (4)

In Equation (4), *k* and *k*_o_ represent the specific rates of solvolysis in a given solvent and in 80% ethanol and *m* is the sensitivity to changes in the original *Y* scale, which was based on the solvolyses of *tert*-butyl chloride [23,28]. They obtained a remarkably good fit, as shown in Equation (5), with a correlation coefficient of 0.992.

log *k* = −3.42 + 0.620*Y* (5)

The −3.42 is the predicted log *k*_o_ value for 80% ethanol (not included in this study) and this can be favorably compared with a subsequently determined [55] experimental value of −3.69. In this subsequent study in 21 hydroxylic solvents, including some containing the highly ionizing 2,2,2-trifluoroethanol (TFE), a correlation using Equation (4) but with *Y*_Cl_ values, based on the specific rates of the bridgehead 1-adamantyl chloride [28,56] (Equation (6)) gave an *m* value of 0.42 ± 0.07. Since there is a good relationship for most of the commonly used solvents between *Y* and *Y*_Cl_, with *Y* = 0.75 *Y*_Cl_ [28], the *m* value would adjust to a value of 0.56 ± 0.09 for a correlation against *Y*, in excellent agreement with the reported [54] value of 0.62.

log (*k/k_o_*) = *mY*_Cl_ (6)

The authors also carried out a Taft Equation treatment involving a consideration of changes in the specific rate of ethanolysis in 100% ethanol as the σ* values, a measure of electronic effects, for the R group in R_2_NCOCl was varied. This type of study is labor intensive since for each point in a Taft Equation treatment a new substrate is required. Accordingly, the substituents were limited to methyl, ethyl, *n*-propyl, isopropyl, and *n*-butyl. The Taft Equation is closely related to the Hammett Equation (Equation (1)). In this instance, the ρσ term is replaced by ρ*σ* and the *k* and *k_o_* relate to aliphatic structures, such as R in R_2_NCOCl in the present case. When R = methyl, σ* is zero by definition and σ* values for the other alkyl groups and for substituted alkyl groups have been determined and tabulated [20,21]. Since steric factors are now also important, a steric parameter E_s_ is included, together with a sensitivity towards changes in the value of E_s_ of δ, to give Equation (7).

log (*k/k_o_*) = ρ*σ* + δE_s_ + *c* (7)

Today, one would carry out a direct multiple regression using readily available user-friendly statistical computation software to obtain the ρ* and δ sensitivities and the constant (residual) term *c*. In 1972 such software was not available and the authors rearranged the equation and carried out a graphical analysis. A graph, given in the Bacaloglu *et al.* manuscript [54], shows plots for both hydrolysis in 50% acetone and ethanolysis, with a grouping of closely related data points separated from the point for R = ethyl. The treatment does not allow the point for R = methyl to be included. The ρ* values estimated were −4.11 for ethanolysis and −3.70 for the hydrolysis in 50% acetone, indicating a partial positive charge on the carbon atom of COCl at the transition state, consistent with an S_N_1 pathway. Caution should be taken as regards the actual values obtained due to the unfortunate spacings and also due to what is generally considered to be an insufficient number of data points for a multiple regression analysis. A review, written by several prominent researchers active in the area of correlation analysis, suggests a *minimum* of five data points for each variable [57], so that this analysis would ideally require double the number of substrates actually studied [54].

When a rigid correlation analysis is carried out, the results show poor correlations with large standard errors for the sensitivity values. For ethanolysis at 25 °C, the values are 0.835 for the multiple correlation coefficient, −7.35 ± 5.32 for ρ* and 0.72 ± 1.75 for δ. For hydrolysis in 50% acetone at 50 °C, the values are 0.907 for the multiple correlation coefficient, −4.61 ± 1.88 for ρ* and 0.78 ± 0.62 for δ. For the hydrolysis in 50% acetone at 20 °C, the values are 0.868 for the multiple correlation coefficient, −7.51 ± 4.07 for ρ* and 1.10 ± 1.34 for δ. It is probably safe to assume that the ρ* is negative but not much else can be deduced from these correlation analyses of the data.

The authors also studied two instances where, instead of the two R groups in R_2_NCOCl, the nitrogen was part of a ring system, in the piperidino- and morpholino-carbamoyl chlorides. In ethanol at 50.0 °C, they arrive at a rate sequence: morpholino (0.60) < Me_2_ (1.00) < *n*-Bu_2_ (2.8) < *n*-Pr_2_ (3.0) < Et_2_ (4.5) < piperidino (8.36) < *i*-Pr_2_ (232). The part of the sequence Et_2_ > *n*-Pr_2_ > *n*-Bu_2_, involving small differences, probably reflect the same sensitivity to changes in steric effects, consistent with the δ value in the Taft treatment of +0.62 for the ethanolysis at 50 °C and of +0.80 for hydrolysis in 50% acetone [54]. The much slower reaction for the 4-morpholinecarbonyl chloride than for the 1-piperidinecarbonyl chloride was considered to reflect the electron-withdrawing influence of the oxygen atom present in the morpholino-derivative. The steric effect suggests either the transition-state being more solvated than the initial molecule or some measure of nucleophilic assistance by the solvent to the primarily S_N_1 process or, of course, a combination of the two effects which will differ only in the extent of actual bond formation at the transition state.

**Figure 1:**
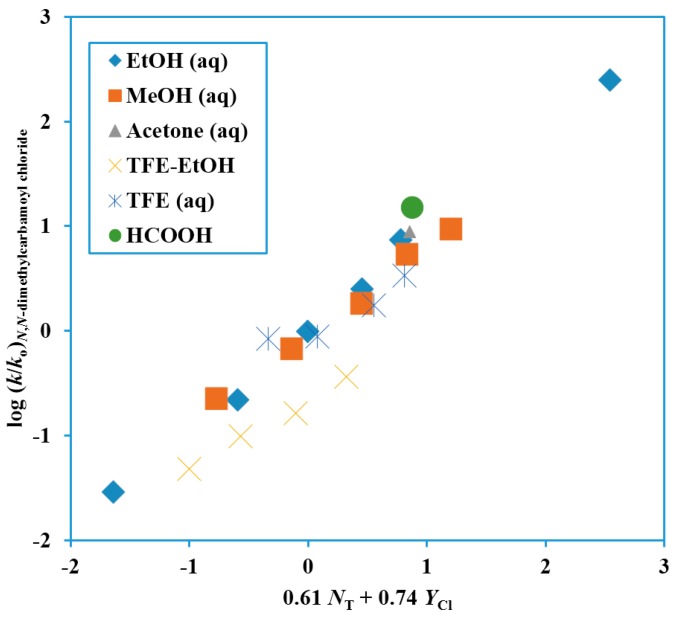
Plot of log (*k*/*k*_o_) for solvolyses of *N*,*N*-dimethylcarbamoyl chloride at 25.0 °C against 0.61 *N*_T_ + 0.74 *Y*_Cl_.

The analyses of the solvolyses of Me_2_NCOCl, which led to an *m* value of 0.42 ± 0.07 for a one-term Grunwald–Winstein equation correlation, gave a much better correlation using the two-term equation (Equation (2)), with use of the *N*_T_ values [29,30] and *Y*_Cl_ values [26,27,28]. Data in 21 solvents, led to the plot shown in Figure 1, with the correlation data shown in Table 1. Both the *l* and *m* values were appreciable with an *l*/*m* ratio of 0.82. This could represent either a loose S_N_2 transition-state or a rather strong assistance from solvation to an ionization process. The observation of common-ion rate depression due to a mass-law effect for hydrolyses in water [40,52] plus the observation that the data point for this hydrolysis lies on the Grunwald–Winstein plot (Figure 1) gives support to the picture involving assistance to an ionization process from solvation.

The determination of product ratios when more than one product results from a reaction can sometimes be helpful in establishing the most probable mechanism. In most cases, some physical measurement (NMR, IR, *etc.*) of the product mixture or a determination after separation (gas or column chromatography, *etc.*) is required. In some instances, the ratio can be very precisely determined by titration and the solvolyses of dialkylcarbamoyl chlorides (but not diarylcarbamoyl chlorides) presents such an opportunity, since the product from water attack decomposes with loss of CO_2_ to give a dialkylamine which is sufficiently basic to neutralize one equivalent of the HCl which is also produced in the solvolysis (Equation (8)). A comparison of the acid produced during alcoholysis with that produced during solvolysis in an alcohol–water mixture gives a direct measure of the product partitioning [55].

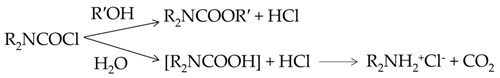
(8)

**Table 1:** Correlation of the first-order rate coefficients for solvolyses of seven disubstituted carbamoyl chlorides (ŔR′′NCOCl) using the original (one-term; Equation (6)) and extended (two-term; Equation (2)) forms of the Grunwald–Winstein equation with use of the *N*_T_ and *Y*_Cl_ scales.

R′	R′′	T °C	*n ^a^*	*l ^b^*	*m ^b^*	*l/m ^c^*	*R ^d^*	References
Me	Me	25.0	6		0.62 *^e^*			[54]
			21		0.42 ± 0.07		0.803	[55]
			21	0.61 ± 0.08	0.74 ± 0.05	0.82	0.958	[55]
			17 *^f^*	0.56 ± 0.05	0.70 ± 0.04	0.80	0.983	[55]
–(CH_2_)_2_–O–(CH_2_)_2_– *^g^*		25.0	20		0.10 ± 0.08		0.297	[58]
			20	0.74 ± 0.10	0.66 ± 0.08	1.12	0.893	[58]
			16 *^f^*	0.74 ± 0.06	0.65 ± 0.05	1.14	0.958	[58]
		35.0	28	0.71 ± 0.05	0.65 ± 0.02	1.09	0.991	[59]
Me	Ph	25.0	32		0.55 ± 0.03		0.965	[60,61]
			32	0.44 ± 0.04	0.68 ± 0.02	0.65	0.994	[60,61]
		60.0	19		0.30 ± 0.04		0.869	[51,61]
			19	0.40 ± 0.08	0.51 ± 0.05	0.78	0.948	[51,61]
Me	*p*-ClC_6_H_4_	25.0	32		0.53 ± 0.04		0.936	[60,61]
			32	0.44 ± 0.06	0.66 ± 0.03	0.67	0.980	[60,61]
Me	*p*-NO_2_CH_4_	25.0	20		0.55 ± 0.09		0.831	[60,61]
			20	0.58 ± 0.06	0.69 ± 0.04	0.84	0.974	[60,61]
1-Ad *^h^*	*p*-CH_3_C_6_H_4_	0.0	11		0.63 ± 0.19		0.745	[62]
Ph	Ph	50.0	10		0.43 ± 0.09		0.864	[63]
		62.5	36		0.48 ± 0.03		0.944	[64]]
		62.5	36	0.23 ± 0.04	0.58 ± 0.03	0.40	0.971	[64]

*^a^* Number of Solvents; *^b^* With accompanying standard error; *^c^* Useful for assigning mechanism. The *l/m* values for phenyl chloroformate of *ca*. 2.96 and for phenyl chlorodithioformate of *ca*. 0.72 [17] are often taken as typical values for addition-elimination reaction, with addition rate-determining, and for ionization with moderate assistance from nucleophilic solvation, respectively; *^d^* Multiple correlation coefficient; *^e^* Calculated using the original *Y* scale, which is based on the first-order rate coefficients for solvolyses of *tert*-butyl chloride; *^f^* With omission of the four 2,2,2-trifluoroethanol (TFE)-ethanol mixed solvents; *^g^* Morpholine with an *N*-chloroformyl substituent; *^h^* The 1-adamantyl group.

The product ratios can be converted to give selectivity values (*S*) using Equation (9).

(9) $$S=\frac{{\left[\text{Ester}\right]}_{\text{solv}}{\left[H_{2}O\right]}_{\text{solv}}}{{\left[\text{Amine}\right]}_{\text{prod}}{\left[\text{EtOH}\right]}_{\text{solv}}}$$

For *N*,*N*-dimethylcarbamoyl chloride solvolyzing in 90%–60% ethanol (remainder H_2_O) the *S* value, at 25.0 °C, was found to be essentially constant at a value of 0.510 ± 0.015 (Table 2). A survey of the literature found almost an exact agreement with a value of 0.512 ± 0.014 for 1-adamantyl nitrate solvolysis at 50.0 °C in the same aqueous ethanol compositions [65]. The solvolyses of the 1-adamantyl nitrate, and 1-adamantyl derivatives in general, are believed to involve product formation at the solvent-separated ion-pair stage [66,67] and the very close similarity gives some support to the concept of this also being the stage for the capture of the dimethylcarbamoyl cation, formed during the ionization of the dimethylcarbamoyl chloride.

**Figure 2:**
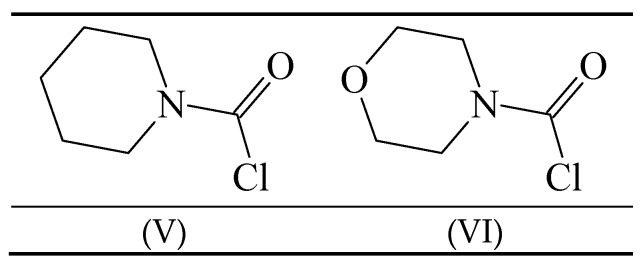
Structures of 1-piperidinecarbonyl chloride (V) and 4-morpholinecarbonyl chloride (VI).

Carbamoyl chlorides in which the “two substituents” on the nitrogen are tied back into a ring have been investigated in terms of mechanism [54,58,59]. The behavior of 1-piperidinecarbonyl chloride (V) (Figure 2) was compared to that of 4-morpholinecarbonyl chloride (VI) in 50% acetone, ethanol and cyclohexanol [54] at temperatures ranging from 20–65 °C for the first two solvents and at the one temperature of 90° for the solvolyses in cyclohexanol. The entropies of activation were at +15.5 and +1.4 in 50% acetone and at −15.9 and −10.2 in ethanol. These intermediate values are not very helpful as regards assigning mechanism.

**Table 2:** Selectivity values (using Equation (9)) for the formation of ester (reaction with alcohol) or amine (reaction with H_2_O) in the solvolyses of *N*,*N*-dimethylcarbamoyl chloride (**1**), 4-morpholinecarbonyl chloride (**2**), and *N*,*N*-diphenylcarbamoyl chloride (**3**).

Solvent ^a^	*S* for 1 *(*25.0 °C*) ^b^*^,*c*^	*S* for 2 *(*25.0 °C*) ^b^*^,*d*^	*S* for 3 (62.5 °C) *^e^*
90% EtOH	0.525	0.666	1.1
80% EtOH	0.510	0.602	0.94
70% EtOH	0.500	0.585	0.82
60% EtOH	0.505	–	0.87
90% MeOH	1.14	1.21	1.4
80% MeOH	1.05	1.13	1.4
70% MeOH	1.04	1.06	1.3
97% TFE	0.230	0.196	0.35
90% TFE	0.334	0.177	0.33
80% TFE	0.356	0.145	0.30 *^f^*

*^a^* On a volume-volume basis, at 25.0 °C, for EtOH–H_2_O and MeOH–H_2_O and on a weight–weight basis for TFE–H_2_O; actual composition is 98.5% of indicated solvent plus 1.5% of acetone; *^b^* Determined by titration of acid produced; *^c^* From [55,58]; *^d^* From ref. [61]; *^e^* Determined by reverse phase HPLC; from [64] (values also listed there for several more aqueous solvent mixtures); *^f^* For 70% TFE.

A comparison can be made of the specific rates of the piperidine and morpholine derivatives. In the text [54], we are told that the morpholine derivative reacts about ten times slower in all three solvolyses. This is consistent with the data reported in their paper [54] for the two alcohols but the data given indicate that, in 50% acetone, the morpholine derivative is the faster by a factor of 2.8 to 3.7. Possibly, there is some presentation error in the entries for the 50% acetone. Assuming S_N_1 reaction, the authors rationalized a slower reaction for the morpholine derivative in terms of the unfavorable electron-withdrawing influence of the oxygen.

A comparison can be made of the specific rates of the piperidine and morpholine derivatives. In the text [54], we are told that the morpholine derivative reacts about ten times slower in all three solvolyses. This is consistent with the data of Table 4 of the paper for the two alcohols but the data given indicate that, in 50% acetone, the morpholine derivative is the faster by a factor of 2.8 to 3.7. Possibly, there is some presentation error in the entries for the 50% acetone. Assuming S_N_1 reaction, the authors rationalized a slower reaction for the morpholine derivative in terms of the unfavorable electron-withdrawing influence of the oxygen.

**Figure 3:**
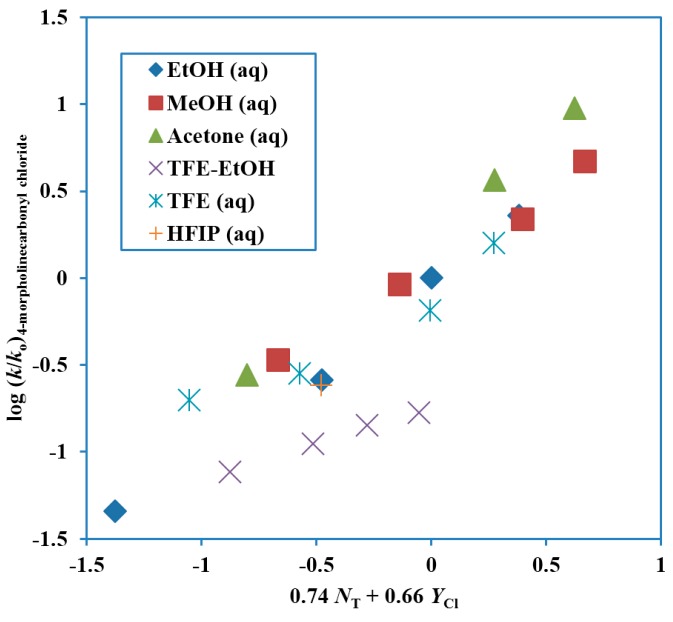
Plot of log (*k*/*k*_o_) for solvolyses of 4-morpholinecarbonyl chloride (4-(chloroformyl)morpholine) at 25.0 °C against 0.74 *N*_T_ + 0.66 *Y*_Cl_.

A study in terms of kinetics and product selectivity values for the 4-morpholinecarbonyl chloride [58] paralleled that for the *N*,*N*-dimethylcarbamoyl chloride [55]. An extended Grunwald–Winstein equation treatment in essentially the same solvents led to values of 0.74 ± 0.06 for *l*, 0.65 ± 0.05 for *m* and −0.05 ± 0.20 for *c*, with an *l*/*m* ratio of 1.14. The plot is shown in Figure 3. The TFE-EtOH values are not included in the reported correlation, which has a correlation coefficient of 0.958. These values are, however, added to the plot, to show the extent of deviation. The correlation values are compared with those for extended Grunwald–Winstein correlations of kinetic data for other substrates in Table 1. The *l*/*m* ratio is similar in value and slightly higher than the 0.80 value for *N*,*N*-dimethylcarbamoyl chloride [55].

The selectivity ratios are very similar to those for the dimethyl derivative, with values slightly higher in ethanol–water mixtures, essentially identical in methanol-water mixtures, and slightly lower in 2,2,2-trifluoroethanol–water mixtures (Table 2). The similarity in behavior is consistent with similar ionization mechanisms, involving appreciable assistance from nucleophilic solvation.

Some fourteen years later, Koo and coworkers [59] revisted the solvolysies of 4-morpholinecarbonyl chloride. They report data at 35.0 °C in a series of solvents very similar to those used at 25.0 °C in the earlier study and arrived at essentially identical values (Table 1) for *l* and *m* of 0.71 ± 0.05 and 0.65 ± 0.02 in an extended Grunwald–Winstein equation treatment and at a value of 0.46 for *m* with the use of the one-term equation. There is no mention of the earlier paper [58], and it seems probable that the different naming system used for the substrate in this earlier paper, naming as 4-(chloroformyl)morpholine, led to it being overlooked. There is, however, an important study of kinetic solvent isotope effects, which is not present in the earlier paper. The ratio of specific rates, *k*_H_/*k*_D_, at 35.0 °C had a value of 1.21 ± 0.02 in methanol and methan(ol-*d*) and of 1.27 ± 0.02 in H_2_O and D_2_O (Table 3). These values are very similar to those observed for reactions believed to proceed by an ionization pathway and considerably lower than those for an addition–elimination pathway, with the addition-step being rate-determining [16,52].

Reactions with *N*,*N*-dialkylcarbamoyl chlorides have usually, for simplicity, been carried out with two identical alkyl groups. Derivatives with different alkyl groups can readily be prepared by reaction of appropriate secondary amines with phosgene [69], and there are instances where such compounds have been found to be synthetically useful. It was found that *N*-*t*-butyl-*N*-alkylcarbamoyl chlorides decomposed on heating in nitrobenzene at 100 °C or in the less polar toluene at higher temperatures to give isobutylene plus HCl together with the alkyl isocyanate, many of which could not be prepared by any of the other techniques available at that time. The favored pathway involved ionization to give a chloride ion followed by its removal of a hydrogen from the *tert*-butyl group, with accompanying formation of isobutylene [69]. This pathway has similarities to that proposed [70] for the decomposition of 1-adamantyl chloroformate on heating in nitrobenzene, when loss of CO_2_ led to 1-adamantyl chloride (Equation (10)).

(10) $$1-\text{AdCOCl}\overset{-{\text{CO}}_{2}}{\to }{\text{1-Ad}}^{+}{\text{Cl}}^{-}\overset{}{\to }\text{1-AdCl}$$

**Table 3:** Kinetic solvent isotope effect (KSIE) for solvolyses of five *N*,*N*-disubstituted carbamoyl chlorides at 25.0 °C.

Substrate	Solvent	*k*_H_/*k*_D_ *^a^*	References
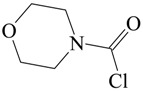	MeOH/MeOD	1.21 ± 0.02	[59]
	H_2_O/D_2_O	1.27 ± 0.02	[59]
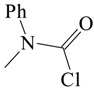	50% MeOH–H_2_O/50% MeOD–D_2_O	1.45 *^b^*	[60]
	H_2_O/D_2_O	1.37 *^b^*	[60]
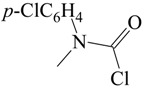	50% MeOH–H_2_O/50% MeOD–D_2_O	1.42 *^b^*	[60]
	H_2_O/D_2_O	1.36 *^b^*	[60]
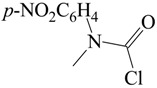	H_2_O/D_2_O	1.43 *^b^*	[60]
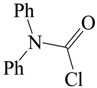	H_2_O/D_2_O	1.17 ± 0.18	[68]

*^a^* Values for *k*_H_/*k*_D_ have been found to be in excess of 1.7 for reactions believed to follow addition-elimination pathways involving general-base catalysis from a second solvent molecule and to be in the region of 1.2 to 1.5 for ionization pathways weakly assisted by nucleophilic solvation ([39,60]); *^b^* Standard deviations (or other error estimates) were not reported for the *k*_H_ and *k*_D_ values.

Despite the difficulty of carrying out a Friedel–Crafts alkylation of nitrobenzene, trace amounts of the *meta*-1-adamantyl-nitrobenzene could be isolated, consistent with the trapping of the very reactive intermediate 1-adamantyl cation. Under solvolytic conditions, the reaction of 1-adamantyl chloroformate led to concurrent solvolysis and decomposition [16,66].

A study was carried out [62] to see whether a parallel pathway would be observed with a 1-adamantyl group on the nitrogen of a carbamoyl chloride. Since the other group was an aryl group, this study will be discussed in the next section of this review.

## 3. Solvolyses of *N*-Alkyl-*N*-arylcarbamoyl Chlorides

The solvolyses of the simplest compound of this type, *N*-methyl-*N*-phenylcarbamoyl chloride was studied in nineteen solvents, including several with a fluoroalcohol component, at 60.0 °C [51]. The higher temperature than for parallel studies of *N*,*N*-dialkylcarbamoyl chlorides reflects a rate reduction on introduction of an aryl group. For example, for ethanolyses at 60 °C, the substrate reacted 17.4 times slower than the *N*,*N*-dimethylcarbamoyl chloride [55], presumably due to the electron-withdrawing influence of the nonconjugated phenyl group hindering the formation of the carbamoyl cation.

An analysis using the extended Grunwald–Winstein equation led to an *l* value of 0.40 ± 0.08 and an *m* value of 0.51 ± 0.05, corresponding to an *l*/*m* ratio of 0.78 ± 0.23 (Table 1). This was suggested to be consistent with an ionization process with appreciable nucleophilic solvation, as was also suggested for the solvolyses of *N*,*N*-dialkylcarbamoyl chlorides [55,58,59]. A consideration of structural, rather than solvent, variations also led to the postulate of an important nucleophilic solvation, which is disrupted by a bulky alkyl group as a substituent on the nitrogen [54].

Subsequently [60], several additional specific rates of solvolysis at 25.0 °C were determined in binary mixtures of water with acetone, ethanol, methanol, TFE and 100% H_2_O. In addition, determinations were made in 100% D_2_O and 50% D_2_O–CH_3_OD. A dissociative S_N_2 transition state was proposed, which is very similar in character, as regards the extent of bond-breaking and bond-making, to the earlier proposed [51] ionization mechanism with appreciable nucleophilic solvation of the developing carbamoyl cation. The specific rates were increased modestly by the incorporation of a *p*-chloro substituent and decreased appreciably by a *p*-nitro substituent, consistent with the proposed developing positive charge on the carbonyl carbon. The relatively small deuterium isotope effects upon the specific rates in H_2_O or D_2_O and in 50% H_2_O–50% MeOH or 50% D_2_O–50% MeOD, with *k*_H_/*k*_D_ values in the range of 1.36 to 1.45 for five comparisons [60] (Table 3) are consistent with either of the proposed (and closely related) mechanisms.

The kinetic data from the two studies [51,60] were combined to allow application of the extended Grunwald–Winstein equation to a reasonably large set of values for specific rates and solvent nucleophilicity and ionizing power parameters [61]. The values obtained are presented together with values from application of the one-term equation (Equation (6)) in Table 1. It can be seen that the two-parameter equation gives much better correlations than the one-term equation, with an *l* value of about 0.5 and *m* value of about 0.7 for the three substrates. The rather low *m* value for an essentially ionization process with moderate nucleophilic solvation probably reflects an internal nucleophilic assistance from the lone pair of electrons on the nitrogen.

**Scheme 2:**
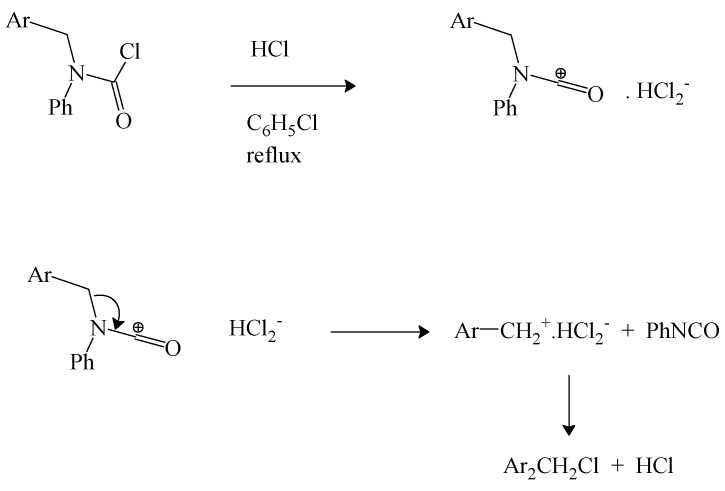
Electrophilic catalysis by HCl.

We have earlier reviewed the decomposition of *N*-*tert*-butyl-*N*-alkylcarbamoyl chlorides to give alkyl isocyanate [69] and the same research group extended this work to a study of *N*-phenyl-*N*-4-isocyanatobenzylcarbamoyl chloride. In refluxing chlorobenzene, in the presence of dry hydrogen chloride, decomposition to 4-isocyanatobenzyl chloride and phenyl isocyanate was observed. It was suggested that the reaction proceeds by an initial protonation at the nitrogen followed by elimination of the benzyl chloride [71]. However, the observation of electrophilic catalysis by FeCl_3_ in the decomposition of the earlier studied [69] *N*-*tert*-butyl-*N*-alkylcarbamoyl chloride suggests that a parallel electrophilic catalysis is a more probable involvement of the added HCl. The coordination of the chloride ion by hydrogen chloride in aprotic solvents to give HCl_2_^−^ anion and the associated catalysis of carbon-halogen bond heterolyses are well established [72]. We propose the pathway shown in Scheme 2.

The solvolyses of *N*-1-adamantyl-*N*-*p*-tolylcarbamoyl chloride have been studied in terms of kinetics and products in alcohols, aqueous alcohols, aqueous acetone and 2,2,2-trifluoroethanol (TFE)-ethanol mixtures [62]. For ethanolyses at 50.0 °C, the rates are intermediate between those for the diethyl and diisopropyl derivatives and it was suggested that an accelerative effect from introduction of a *tert*-alkyl group was counterbalanced by a retarding effect on introducing the aryl group.

A correlation analysis using the two-term Grunwald–Winstein equation (Equation (2)) indicated only a low sensitivity to changes in solvent nucleophilicity and the specific rates could adequately be correlated using the one-term equation (Equation (6)), with a sensitivity to solvent ionizing power (*m*) of 0.63 ± 0.19 (Table 1).

Product studies (gas chromatography) did not find any alcohol, ether, or chloride derived from adamantane after solvolyses in aqueous ethanol or aqueous acetone. In 1,1,1,3,3,3-hexafluoro-2-propanol (HFIP), after 5 min at 25 °C, the products included 31% of the ether and 34% of 1-adamantyl chloride. In the highly ionizing TFE, but less so than HFIP, after two days, 86% of the ether and 14% of 1-adamantyl chloride was detected. It was proposed that in aqueous ethanol or acetone the carbamoyl cation, formed in an S_N_1 process, reacted sufficiently rapidly with the nucleophilic components of the solvent that loss of aryl isocyanate was not observed. In the considerably less nucleophilic HFIP and TFE, loss of *p*-tolyl isocyanate becomes competitive and the 1-adamantyl cation formed is then captured either by a nucleophilic component of the solvent, to give an ether, or by a union with the chloride counter ion (Equation (11)).


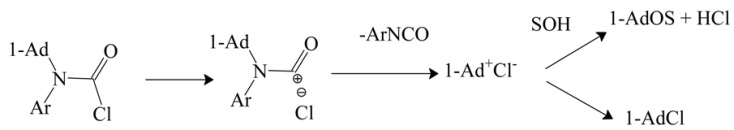
(11)

These observations suggest that the loss of an isocyanate (Ar–N=C=O) from a carbamoyl cation (1-Ad–N(Ar)–C=O)^+^ is considerably less facile than the loss of carbon dioxide (O=C=O) from a carboxylium ion (1-Ad–O–C=O)^+^. This is consistent with the appreciable stability of the parent carbamoyl chloride (H_2_NCOCl) [2,3,4] relative to the highly unstable formyl chloride (HOCOCl) [1].

## 4. Solvolyses of *N*,*N*-Diarylcarbamoyl Chlorides

A kinetic study by Johnson and Giron of the hydrolysis of *N*,*N*-diphenylcarbamoyl chloride [68,73] concluded that an ionization (S_N_1) pathway was dominant. Supporting this claim was a slightly above unity *k*_H_2_O_/*k*_D_2_O_ ratio of 1.2 at 25.0 °C. This value is much lower than values >2 which are observed when general-base catalysis assists a rate-determining addition in an addition-elimination pathway for substitution at a carbonyl carbon [73]. Further support came from an only slightly negative (−5 cal·mol^−1^·K^−1^) entropy of activation, consistent with a unimolecular pathway. The structure at the transition state was considered to be loose and the solvolysis mechanism to be “nearly unimolecular”. The solvolyses were found to be insensitive to addition of several nucleophiles but addition of amines led to a competitive pathway. The tendency to unimolecular reaction was considered to be less extreme that for *N*,*N*-dimethylcarbamoyl chloride which had a slightly positive entropy of activation (+5 cal·mol^−1^·K^−1^) and which showed a lack of reaction with added amines. The reactions are appreciably slower than with either two methyl groups or a methyl and phenyl group on the nitrogen. Relative ratios are Ph_2_NCOCl (1.0), PhMeNCOCl (46), Me_2_NCOCl (800) for the ethanolyses at 60.0 °C [51].

The F/Cl leaving-group rate ratio can be an excellent probe for deciding between ionization mechanisms and addition-elimination pathways, with addition rate-determining. When the carbon-halogen bond is not being broken in the rate-determining step but is only being subjected to hybridization changes, then the ratio is close to unity and frequently it is even found to be somewhat above unity. For ionization pathways, the much stronger carbon-fluorine bond leads to a very small *k*_F_/*k*_Cl_ rate ratio. For example, for the 1-adamantyl haloformates solvolyzing in methanol or ethanol the *k*_F_/*k*_Cl_ rate ratio is about 10^−5^ [66]. Sometimes, the ionization pathway for the fluoroformate is so slow that by default the addition-elimination pathway becomes the dominant one and small values for the ratio but less small that the ratio if both solvolyzed by ionization, are observed. The application to the solvolyses of haloformates has recently been reviewed [16]. Applying to the *N*,*N*-diphenylcarbamoyl halides, for hydrolysis in 90% water-10% acetonitrile at 25.0 °C, led to a *k*_F_/*k*_Cl_ ratio of 2.1 × 10^−3^ [68], consistent with an ionization mechanism but considerably higher than the value of about 10^−6^ for the hydrolyses of the *N*,*N*-dimethylcarbamoyl halides [74]. Possibly, the hydrolyses of the *N*,*N*-diphenylcarbamoyl fluoride is to a large degree addition-elimination in character.

Kim, Song, and Lee also proposed an S_N_1 mechanism for solvolyses of Ph_2_NCOCl but very questionable is their assignment of an S_N_2 mechanism for (CH_3_)_2_NCOCl solvolyses. Possibly, they were just emphasizing that there is evidence for more nucleophilic assistance from the solvent for the solvolyses of the dialkyl compound [75].

**Figure 4:**
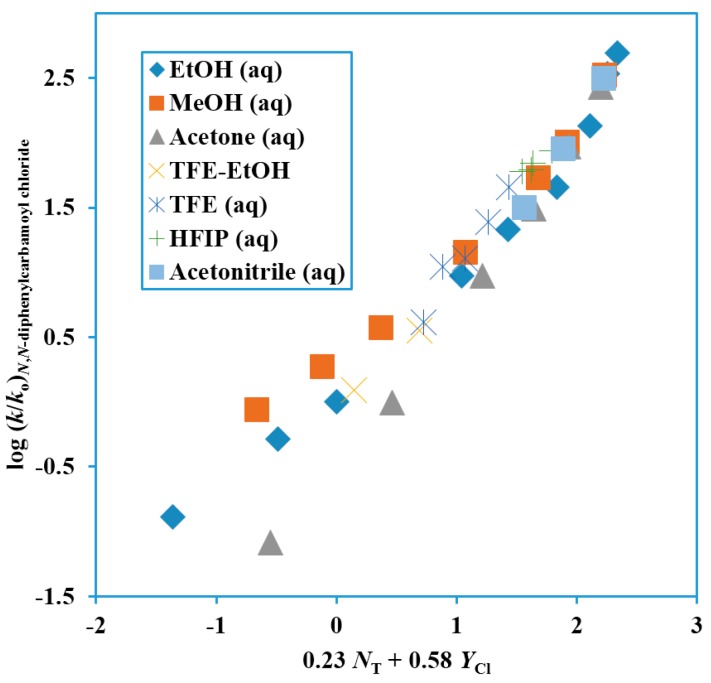
Plot of log (*k*/*k*_o_) for solvolyses of *N*,*N*-diphenylcarbamoyl chloride at 62.5 °C against 0.23 *N*_T_ + 0.58 *Y*_Cl_.

Subsequently, a kinetic study of the solvolyses of Ph_2_NCOCl at 62.5 °C was augmented by fourteen values obtained by extrapolation of data obtained at lower temperatures [68,75]. A total of 36 hydroxylic solvents were used for the correlation of the solvolyses using the one-and two-term Grunwald–Winstein equation [64]. The values from the correlations are tabulated in Table 1 and the two-term plot is shown as Figure 4.

This study [64] also considered the selectivity for product formation on replacing the chlorine by the alkoxy or hydroxyl components from within a mixed binary hydroxylic solvent. Unlike the situation for *N*-*N*-dialkyl compounds, the presence of one or two aryl groups on the nitrogen severely reduces the basicity of the corresponding secondary amine, which, when released in the solvolyses process, no longer neutralizes an equivalent amount of the acid formed. Therefore, selectivity values can no longer be obtained by titration and the product portioning was determined by reversed-phase HPLC. The selectivity values (*S*) defined as for reaction with alcohol (ROH) relative to reaction with water in aqueous alcohol mixtures are obtained using Equation (12), with the diphenylamine resulting from the rapid Ph_2_NCOOH → Ph_2_NH + CO_2_ reaction.

(12) $$S=\frac{\left[{Ph}_{2}NCOOR]\;[H_{2}O\right]}{\left[{Ph}_{2}NH]\;[ROH\right]}$$

The *S* values in aqueous ethanol are almost constant with a value of 1.1 (90% ethanol) decreasing to 0.78 (40% ethanol) and then rising again to 0.94 (20% ethanol). The values for aqueous methanol are at a value of 1.4 ± 0.1 for the full range of 90%–10% methanol. In aqueous TFE a value of 0.33 ± 0.03 in observed across a range of 97%–50% TFE (Table 2). These values are similar to those for *p*-methoxybenzoyl chloride, also considered to solvolyze by a mechanism close to S_N_1 but with some nucleophilic assistance [76,77].

There has also been discussion [61] as to whether the correlation analyses of the specific rates of solvolysis of Ph_2_NCOCl are improved by addition of a relatively small contribution from a third-term governed by the aromatic ring parameter (*I*) [31].

Liu and coworkers [63] have also studied the solvolyses of *N,N*-diphenylcarbamoyl chloride. Earlier, this research group had developed *Y*_BnCl_ [78] and *Y*_xBnCl_ scales [79] for application to the solvolyses of benzyl derivatives and benzhydryl (or 9-fluorenyl) derivatives, respectively. These scales differ somewhat, but not enormously, from the original *Y*_Cl_ scale [28,56]. In this paper, these scales were applied to the solvolyses of Ph_2_NCOCl, and it was proposed that the carbamoyl cation formed in an ionization process can be represented by a resonance hybrid structure which includes contributing structures with a phenyl cation associated with phenyl isocyanate. One can draw such structures, but one would expect that such a contribution, containing a high energy phenyl cation, would make a negligible contribution to the overall hybrid. It is mentioned that experimental difficulties limited the study to ten solvents. Since the application of the Grunwald–Winstein equation with *N*_T_ together with either *Y*_BnCl_ (*l* = 0.22 ± 0.06; *m* = 0.51 ± 0.06) or *Y*_xBnCl_ (*l* = 0.21 ± 0.07; *m* = 0.54 ± 0.08) gives essentially the same parameters as when *N*_T_ and *Y*_Cl_ are used with 36 solvents (Table 1), one can conclude both that the correlations for solvolyses of Ph_2_NCOCl are robust and that there is nothing to be gained by substituting *Y*_BnCl_ or *Y*_xBnCl_ for *Y*_Cl_ in these correlations.

## 5. Reactions of Monosubstituted and Unsubstituted Carbamoyl Chlorides

There have been very few mechanistic studies involving monosubstituted and unsubstituted carbamoyl chlorides. Monosubstituted derivatives can be prepared in good yields, in a solvent such diphenyl ether, at temperatures elevated to 240 °C by reaction of an alkylamine with phosgene [80]. Distilling the product with an amine, such as *N*,*N*-dimethylaniline, results in loss of HCl and formation of the isocyanate (Equation (13)).


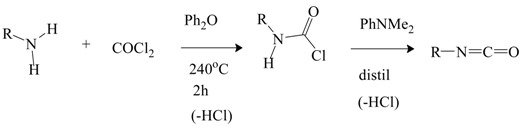
(13)

In a parallel study [81], aromatic isocyanates are prepared by first reacting an aromatic monoamine with COCl_2_ below the decomposition point of the aromatic carbamoyl chloride and then raising the temperature.

The kinetics of the parent and three substituted phenylcarbamoyl chlorides in terms of liberation of HCl and formation of the isocyanate have been studied [82] as a 1% solution in toluene in the range of 40–80 °C. Activation energies in the range of 13.2 to 14.7 kcal·mol^−1^ were observed.

The decomposition, in acetonitrile as solvent at 25.0 °C, of arylcarbamoyl chlorides was studied in terms of both equilibrium and rate by Bacaloglu and Bunton [83]. Surprisingly, there was essentially no effect on the specific rate of decomposition to aryl isocyanate and HCl when the ring-substituent was varied from one which was strongly electron supplying (4-MeO) to one which was strongly electron withdrawing (3-NO_2_). As regarding the equilibrium constant for the process (Equation (14)), the values varied from 9.6 × 10^−3^ to 3.0 × 10^−3^ mol·L^−1^ over the same range of substituents. For the parent and ten ring-substituted derivatives, a good plot against the Hammett σ^−^ values [19,20,21,22] was obtained with a slope (ρ value) of −0.52, indicating a modest increase in the amount of carbamoyl chloride at equilibrium in the presence of electron-withdrawing substituents. 


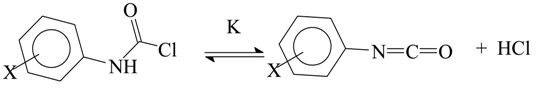
(14)

The 2,6-diethyl derivative (both *ortho*-positions occupied) gave appreciable increases in acetonitrile in both the equilibrium and rate constants, consistent with a reduction in the steric interactions on progressing to the isocyanate.

It would be worthwhile studying these reactions under solvolytic conditions. The interaction of the HCl with a hydroxylic solvent would seriously hinder any reverse reaction for the reaction of Equation (14) and the obtaining of specific rates for the forward reaction within the equation should be simplified (Equation (15)).

The same authors also studied the effects of the addition of an aromatic amine to the reactions [84]. The amine-promoted reactions initially gave aryl isocyanate which subsequently reacted with excess amine, if primary or secondary, to give *N,N*-diarylureas. The situation is quite complex with differing reaction pathways being proposed for electron-attracting and electron-withdrawing substituents. For more details, the reader is referred to the original publication [84].

There have been very few reports concerning the preparation and reaction of the parent (unsubstituted) carbamoyl chloride. It is noteworthy that, unlike with formyl chloride (HOCOCl), this does not seem to be due to a lack of stability. Two reports of its synthesis both involve reactions occurring in the 350–400 °C range. One industrial synthesis [3] involved treatment of urea with dry HCl gas in the presence of granular sand at 350 °C, followed by cooling to 250 °C to remove sold NH_4_Cl and then collection of the liquid carbamoyl chloride in a water-cooled condenser.


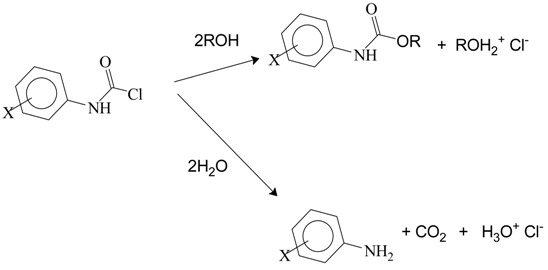
(15)

A smaller scale synthesis involved a direct formation from ammonia and COCl_2_ at about 400 °C, with a yield of 90%–95%. It could be stabilized, prior to subsequent reaction, by forming a molecular complex with AlCl_3_ or FeCl_3_. These complexes can then be reacted with aromatic hydrocarbons to give acid amides in about 90% yields [2]. Olah [85] has investigated the complexes formed with Lewis Acids (such as with AlCl_3_ or FeCl_3_ [2]) by carbamoyl chlorides or fluorides and found that the complexing involves coordination with the carbonyl oxygen. Similarly, the protonation of carbamoyl fluorides, including the parent, by FSO_3_H or FSO_3_H-SbF_5_ also takes place at the carbonyl oxygen.

There has been a theoretical study of the reactions of carbamoyl and thiocarbamoyl halides (chlorides and fluorides) with ammonia in either the gas phase or in solution [86]. It was concluded that the mechanisms resemble those for acetyl and thioacetyl halides [87] but the resonance stabilization of the carbamoyl and thiocarbamoyl groups leads to a lower reactivity for their chlorides or fluorides. Upon transfer from the gas phase to aqueous solution, it was assumed that only solvation considerations would be introduced and the possibility of an intervention by water molecules changing the reaction pathway was not considered within the theoretical treatment.

Heterolytic bond dissociation energies (kcal·mol^−1^, gas phase), calculated by G3 MO theory [88], show strong substituent effects: H_2_NCOCl (169.1), MeNHCOCl (161.9), Me_2_NCOCl (152.2), H_2_NCSCl (152.0) and Me_2_NCSCl (138.0); the latter three are much lower than for MeCOCl (161.8). These numbers combine the effects of ground state stabilization and cation stabilization, and also show that H_2_NCOCl is relatively stable (as observed).
